# Risk of new-onset seizures following immunization against COVID-19: a self-controlled case-series study

**DOI:** 10.4178/epih.e2025024

**Published:** 2025-05-02

**Authors:** Hwa Yeon Ko, Dongwon Yoon, Ju Hwan Kim, Han Eol Jeong, Seung Bong Hong, Won-Chul Shin, Ju-Young Shin, on behalf of the CoVaSC Investigators

**Affiliations:** 1 School of Pharmacy, Sungkyunkwan University, Suwon, Korea; 2 Department of Biohealth Regulatory Science, Sungkyunkwan University, Suwon, Korea; 3 Department of Neurology, Samsung Medical Center, Seoul, Korea; 4 Sungkyunkwan University School of Medicine, Seoul, Korea; 5 Department of Neurology, Kyung Hee University Hospital at Gangdong, Kyung Hee University College of Medicine, Seoul, Korea; 6 Department of Clinical Research Design & Evaluation, Samsung Advanced Institute for Health Sciences & Technology, Sungkyunkwan University, Seoul, Korea

**Keywords:** Vaccines, Seizures, Safety, COVID-19, Immunization programs

## Abstract

**OBJECTIVES:**

Despite emerging reports of new-onset seizures (NOS) following coronavirus disease 2019 (COVID-19) vaccination, safety evidence regarding the risk of NOS after vaccination remains limited. We aimed to investigate the potential association between NOS and COVID-19 vaccination.

**METHODS:**

We conducted a self-controlled case series study utilizing a nationwide database linking the COVID-19 vaccination registry and the National Health Information Database (from February 2021 to October 2022). We identified adults (≥18 years) who received COVID-19 vaccination (BNT162b2, ChAdOx1 nCoV-19, mRNA-1273, NVX-CoV2373, or Ad26.COV2.S) and had a diagnosis of NOS accompanied by prescriptions of anti-seizure drugs. The observation period was defined as 240 days following vaccination. We evaluated the risk of NOS during a risk window of 28 days after vaccination compared to the control window (the remaining observation period excluding the risk window). Incidence rate ratios (IRRs) with 95% confidence intervals (CIs) were estimated using a conditional Poisson regression model.

**RESULTS:**

Among 42,155,198 COVID-19 vaccine recipients, we identified 1,849 and 4,217 patients with NOS in the risk and control windows, respectively. There was no increased risk of NOS within the 28-day period following vaccination (IRR, 0.99; 95% CI, 0.94 to 1.05). Although results from subgroup analyses by vaccine type were largely consistent with the main findings (IRR, 0.95; 95% CI, 0.88 to 1.03 for BNT162b2; IRR, 0.95; 95% CI, 0.77 to 1.16 for ChAdOx1 nCoV-19; IRR, 1.58; 95% CI, 0.52 to 4.83 for Ad26.COV2.S), a marginally elevated risk was observed for mRNA-1273 (IRR, 1.21; 95% CI, 1.04 to 1.42).

**CONCLUSIONS:**

There was no evidence of an increased risk of NOS following COVID-19 vaccination. These findings can be used as safety evidence in clinical decision-making and to bolster public confidence in COVID-19 vaccines.

## GRAPHICAL ABSTRACT

[Fig f4-epih-47-e2025024]

## Key Message

• A nationwide self-controlled case series study investigated whether COVID-19 vaccination increases the risk of new-onset seizures (NOS) in adults by analyzing over 42 million vaccine recipients.

• The results showed no increased risk of NOS within 28 days after vaccination for most vaccine types, with the exception of a marginally elevated risk for the mRNA-1273 vaccine.

• Overall, the study found no evidence of increased NOS risk following COVID-19 vaccination, supporting the safety of these vaccines for clinical decision-making.

## INTRODUCTION

Since the emergence of coronavirus disease 2019 (COVID-19), over 13.3 billion doses of COVID-19 vaccines have been administered worldwide [1]. The implementation of this mass vaccination campaign has been crucial not only for preventing COVID-19 infections but also for improving the long-term management of COVID-19-related disease [2-4]. Although COVID-19 vaccines are recognized as highly effective in preventing infection and its post-acute sequelae, numerous cases of new-onset seizures (NOS) following COVID-19 vaccination continue to be reported among diverse ethnicities, age groups, and vaccine types [5-12]. These ongoing reports undermine public trust in vaccines, highlighting the importance of evaluating the association between COVID-19 vaccines and NOS.

While the precise biological mechanisms behind vaccine-induced seizures remain unidentified, plausible explanations include immune-mediated abnormal neuronal activity [13] or seizures triggered by fever due to a sudden increase in body temperature [14]. Alternatively, seizures could occur secondary to other neurological adverse events, such as ischemic stroke, encephalitis, or cerebral venous sinus thrombosis following vaccination [8]. Given that COVID-19 infection itself is a known risk factor for seizures [15], well-designed studies are required to differentiate the effects of COVID-19 vaccination from those of infection to accurately assess the relationship with NOS. Previous observational studies addressing this clinical concern have either lacked sufficient statistical power due to small sample sizes or were limited to assessing only a few vaccine types [16-18].

To address this evidence gap, the current study aimed to assess the association between the risk of NOS and immunization against COVID-19 using a self-controlled case series (SCCS) analysis based on a nationwide Korean database that includes multiple COVID-19 vaccine types.

## MATERIALS AND METHODS

### Data source

This study was conducted as part of Korea’s COVID-19 Vaccine Safety Research Committee (CoVaSC), contributing to the generation of evidence supporting the safe use of COVID-19 vaccines. CoVaSC investigators use a large, linked database combining the COVID-19 vaccination registry (from February 26, 2021, to October 31, 2022), managed by the Korea Disease Control and Prevention Agency (KDCA), and healthcare claims data (from January 1, 2002, to October 31, 2022) provided by the National Health Insurance Service (NHIS) [19]. In Korea, vaccine distribution is government-managed, enabling KDCA to systematically collect vaccination records for the entire Korean population since the initiation of COVID-19 vaccinations (February 26, 2021). These records include vaccination dates, doses, and the specific vaccine types administered. The NHIS, serving as a single-payer insurer, covers the entire Korean population (>50 million people). Comprehensive healthcare utilization data, including medical diagnoses, drug prescriptions, and medical examinations, are anonymized via personal identifiers and made accessible to researchers. By linking claims data to the COVID-19 vaccination registry, a large-scale data source was established to identify adverse events among vaccine recipients. Diagnostic records are coded according to the International Classification of Diseases, 10th revision (ICD-10), and prescription drug information is identified using domestic drug chemical codes based on the Anatomical Therapeutic Chemical (ATC) classification of the World Health Organization.

### Study population

**Figure 1.**
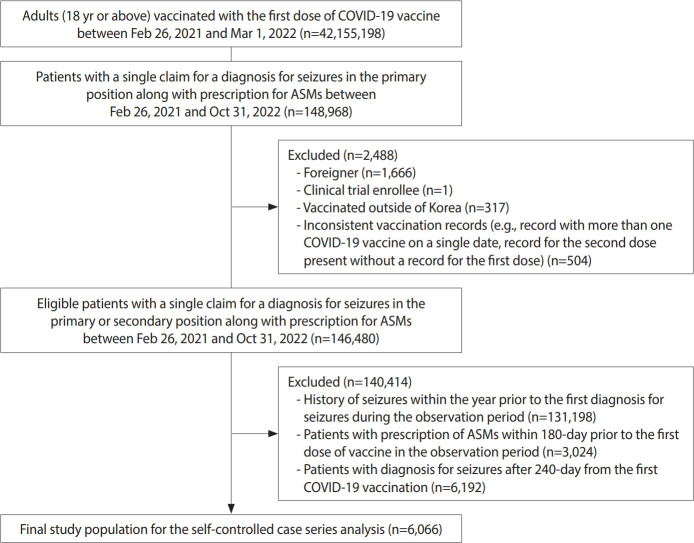
Flowchart of study participant selection. COVID-19, coronavirus disease 2019; ASMs, anti-seizure medications.

We identified adults aged 18 years or older who received their first dose of the COVID-19 vaccine between February 26, 2021, and March 1, 2022. Among these, individuals diagnosed with seizures recorded as the primary diagnosis and prescribed anti-seizure medications (ASMs) between February 26, 2021, and October 31, 2022, were included. As the study employed a self-controlled case series design, only patients experiencing both the exposure and the outcome of interest within the predefined observation period were eligible for inclusion. We excluded participants who were foreigners, enrolled in clinical trials for COVID-19 vaccines, vaccinated abroad, or those with vaccination records deviating from the recommended COVID-19 vaccination guidelines (e.g., more than 1 vaccine type administered on the same day, or a record for the second dose without documentation of the first dose). Additionally, individuals diagnosed with seizures in the year prior to their first seizure diagnosis during the predefined observation period, and those prescribed ASMs within 180 days prior to the date of their first COVID-19 vaccination, were excluded to focus exclusively on incident cases. Patients diagnosed with seizures more than 240 days after the first vaccine dose were also excluded. The flowchart illustrating the selection process for study participants is presented in Figure 1.

### Study design

We employed a SCCS design [20] to evaluate the risk of NOS following COVID-19 vaccination. This choice was motivated by the high COVID-19 vaccine exposure rates within the Korean population, which rendered it challenging to select appropriate controls receiving different vaccines. The SCCS design estimates the relative incidence between risk and control windows within the same individual. Since each individual serves as their own control, this design inherently minimizes confounding from time-invariant covariates, such as sex and genetic factors. As presented in Supplementary Material 1, the observation period was specified as 240 days following the first vaccine dose. Within this period, intervals up to 28 days after each vaccination dose were designated as risk windows, during which each individual is presumed to have a higher risk of the outcome due to vaccination. The 28-day risk window was chosen based on evidence from clinical trials indicating that neutralizing antibodies against severe acute respiratory syndrome coronavirus 2 (SARS-CoV-2) peak at approximately 28 days post-vaccination [21,22]. The remaining observation periods outside these risk windows served as control windows.

### Vaccination against coronavirus disease 2019

All identifiable doses (first, second, third, fourth) and types of COVID-19 vaccines administered were considered exposures. The following vaccine types were available in Korea during the study period: BNT162b2 (Pfizer-BioNTech), ChAdOx1 nCoV-19 (AstraZeneca), mRNA-1273 (Moderna), Ad26.COV2.S (Janssen), and NVX-CoV2373 (Novavax). The recommended intervals for the primary series between the first and second doses were 28 days for mRNA-1273 and ChAdOx1 nCoV-19, and 21 days for BNT162b2 and NVX-CoV2373. A single dose of Ad26.COV2.S constituted a complete primary series. Individuals were categorized as receiving a homologous vaccine series if they received the same COVID-19 vaccine type for all doses, or heterologous if they received different vaccine types for any of the doses.

### Outcome

The outcome of interest was NOS, defined as a seizure diagnosis recorded as the primary diagnosis in either inpatient or outpatient settings, accompanied by a prescription for ASMs on the same day. Seizure diagnoses were identified using ICD-10 codes (G40, G41, R56, F44.5), and ASM prescriptions were identified by ATC codes (N03A) (Supplementary Material 2). Recurrent seizures were not included as outcomes, and only the first seizure occurrence was considered to avoid violating the assumptions inherent to the SCCS design [20]. We observed all NOS events occurring during the predefined observation period among eligible participants, and calculated incidence rates within the designated risk and control windows.

### Statistical analysis

Descriptive statistics were used to summarize NOS cases, categorized by whether they occurred within risk or control windows, according to baseline characteristics such as age, sex, residence, comorbidities, and vaccine doses or types administered. The t-test was conducted for continuous variables, and the chi-square test was utilized for categorical variables. A p-value of less than 0.05 was considered indicative of statistical significance. For the primary analysis, conditional Poisson regression models were used to estimate the number of events, person-years (PYs), incidence rate ratios (IRRs), and corresponding 95% confidence intervals (CIs) to evaluate the risk of NOS following vaccination. Secondary analyses included stratified evaluations by vaccine doses (first, second, third, fourth, or first/second combined), vaccination strategies (heterologous or homologous), and vaccine platforms (mRNA or non-mRNA).

We also conducted subgroup analyses stratified by age group (18-29, 30-39, 40-49, 50-59, 60-69, 70-79, and ≥80 years), sex (male, female), insurance type (health insurance, Medical Aid), residential area (metropolitan, rural), Charlson comorbidity index score (<5, ≥5), and history of specific comorbidities recorded during the year preceding the first vaccination dose. These comorbidities included myocardial infarction, congestive heart failure, peripheral vascular disease, stroke, dementia, chronic pulmonary disease, rheumatic disease, peptic ulcer disease, mild liver disease, diabetes mellitus, diabetic complications, hemiplegia/paraplegia, renal disease, cancer, serious liver disease, solid or metastatic tumors, and human immunodeficiency virus infection.

Several sensitivity analyses were additionally performed. First, we repeated the main analysis using alternative risk windows (14 and 42 days) to evaluate how different timeframes influenced NOS risk estimates. Second, individuals who died within 7 days after the onset of NOS were excluded to remove potentially susceptible cases. Third, individuals infected with COVID-19 before their first vaccination date or before their NOS diagnosis date were excluded, considering that COVID-19 infection itself might elevate seizure susceptibility [15]. Fourth, we employed a stricter definition of NOS by restricting the diagnosis to those identified through inpatient or emergency room visits only, thereby minimizing outcome misclassification. Lastly, we conducted a post-hoc analysis specifically among individuals vaccinated with mRNA-1273. All statistical analyses were performed using SAS Enterprise Guide version 8.3 (SAS Institute Inc., Cary, NC, USA).

### Ethics statement

This study received approval from the Public Institutional Review Board Designated by Ministry of Health and Welfare (P01-202203-01-005) and adhered to the ethical principles outlined in the Declaration of Helsinki. Given the utilization of anonymized claims data in this study, the requirement for informed consent was waived. This study followed the Strengthening the Reporting of Observational Studies in Epidemiology (STROBE) guideline for cohort studies (Supplementary Material 3).

## RESULTS

A total of 129,956,027 COVID-19 vaccine doses were administered in Korea during the study period. Among the 42,155,198 adults who received their first dose of a COVID-19 vaccine between February 26, 2021, and March 1, 2022, we identified 1,849 patients who experienced NOS within the designated risk window following vaccination. The mean age of the 1,849 patients who experienced NOS in the risk window was 54.6 years (standard deviation [SD], 19.7), while the mean age of the 4,217 patients who experienced NOS in the control window was 57.0 years (SD, 19.9). Women represented 768 (41.5%) of patients in the risk window and 1,800 (42.7%) of patients in the control window. Regarding comorbidities assessed during the year prior to the first vaccine dose, the Charlson comorbidity index was significantly higher in patients experiencing NOS in the control window (mean±SD, 1.9±2.3) compared with those in the risk window (mean±SD, 1.7±2.2). Patients in the control window also had a higher prevalence of dementia (n=715, 17.0% in the control window; n=255, 13.8% in the risk window) and chronic pulmonary disease (n=739, 17.5%; n=284, 15.4%). Diabetes mellitus (n=951, 22.6% in the control window; n=384, 20.8% in the risk window) and mild liver disease (n=934, 22.1%; n=394, 21.3%) were each prevalent in over 20% of patients experiencing NOS (Table 1).

**Figure 2.**
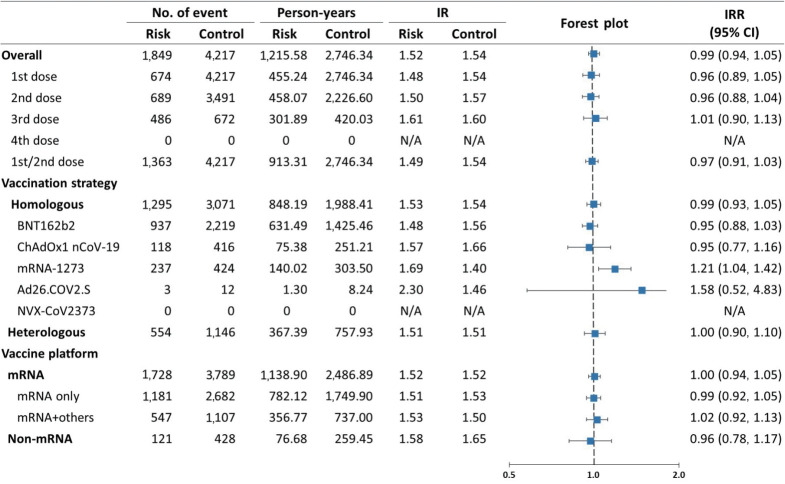
Risk of new-onset seizures within 28 days following coronavirus disease 2019 vaccination, overall and stratified by vaccine doses, vaccination strategies, and vaccine platforms. IR, incidence rate; IRR, incidence rate ratio; CI, confidence interval; N/A, not available.

As presented in Figure 2, the incidence of NOS was 1.52 per PY in the risk window and 1.54 per PY in the control window. The incidence of NOS increased gradually with each subsequent vaccine dose in both risk and control windows. Overall, there was no increased risk of NOS within 28 days following COVID-19 vaccination (IRR, 0.99; 95% CI, 0.94 to 1.05). Results from analyses stratified by first (IRR, 0.96; 95% CI, 0.89 to 1.05), second (IRR, 0.96; 95% CI, 0.88 to 1.04), third (IRR, 1.01; 95% CI, 0.90 to 1.13), and primary series doses (first and second doses combined; IRR, 0.97; 95% CI, 0.91 to 1.03) were consistent with the main analysis. Although subgroup analyses by vaccine type generally aligned with the main results (IRR, 0.95; 95% CI, 0.88 to 1.03 for BNT162b2; IRR, 0.95; 95% CI, 0.77 to 1.16 for ChAdOx1 nCoV-19; IRR, 1.58; 95% CI, 0.52 to 4.83 for Ad26.COV2.S), a marginally elevated risk was noted for recipients of mRNA-1273 (IRR, 1.21; 95% CI, 1.04 to 1.42). Post-hoc analyses among recipients of homologous mRNA-1273 vaccination indicated no increased risk for the first (IRR, 1.01; 95% CI, 0.79 to 1.29) or third doses (IRR, 1.29; 95% CI, 0.92 to 1.80), although a significantly elevated risk was observed after the second dose (IRR, 1.35; 95% CI, 1.09 to 1.67) (Supplementary Material 4).

**Figure 3.**
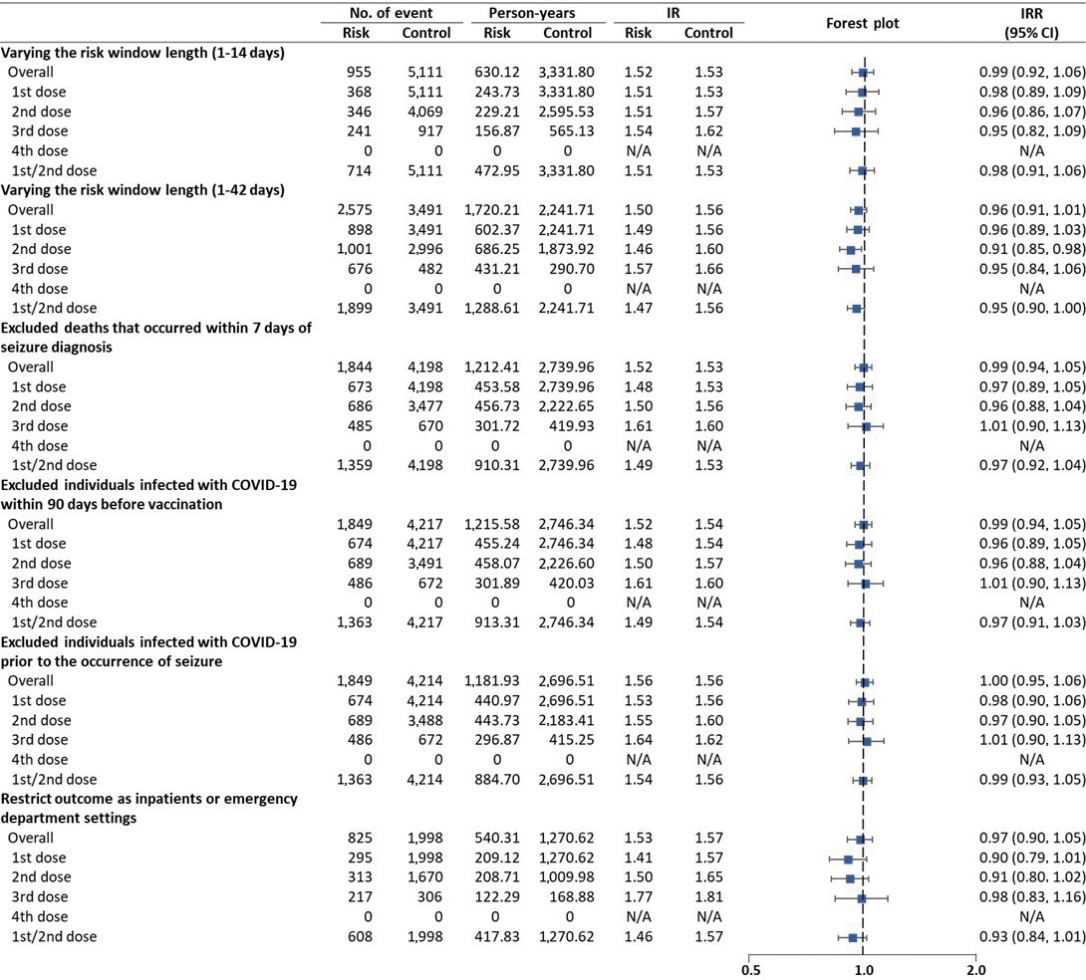
Risk of new-onset seizures following coronavirus disease 2019 vaccination in the sensitivity analyses. IR, incidence rate; IRR, incidence rate ratio; CI, confidence interval; N/A, not available.

Subgroup analyses stratified by age group, sex, insurance type, region, and comorbidities generally showed results consistent with the primary findings, indicating no increased NOS risk following vaccination. However, the age group of 30-39 years exhibited a slightly increased risk of NOS (IRR, 1.31; 95% CI, 1.10 to 1.55) (Table 2). All sensitivity analyses yielded findings consistent with the primary analysis (Figure 3).

## DISCUSSION

This nationwide study found no increased risk of NOS within 28 days following COVID-19 vaccination. The main results were consistent across subgroup analyses categorized by age group, sex, vaccine dose, and underlying comorbidities. The robustness of these findings was further confirmed through multiple sensitivity analyses, including varying the risk window duration, restricting participants without COVID-19 infections, and applying a more stringent outcome definition.

To our knowledge, this study represents the largest population-based evaluation of the risk of NOS following COVID-19 vaccination conducted to date. Our findings align with those of a previous SCCS study from Hong Kong, which included 426 and 263 incident seizure cases following vaccination with BNT162b2 and CoronaVac, respectively [16]. That study reported no increased seizure risk during days 1 to 6 following BNT162b2 (IRR, 1.39; 95% CI, 0.75 to 2.58) or CoronaVac (IRR, 1.19; 95% CI, 0.50 to 2.83). Similarly, another SCCS study conducted in Malaysia found no significant association between vaccination with CoronaVac (IRR, 1.15; 95% CI, 0.91 to 1.44) or ChAdOx1-nCoV-19 (IRR, 1.47; 95% CI, 0.83 to 2.59) and seizure risk within a risk window of 1-21 days after vaccination [18]. Both previous studies assessed periods during which the B.1.617.2 (Delta) variant was dominant (before January 2022 in Korea [24]). Our findings support these prior studies with reassuring results covering the period when the B.1.1.529 (Omicron) variant was dominant (after January 2022), further reinforcing the safety profile across diverse types and doses of COVID-19 vaccines in a larger population, with more precise risk estimates.

The biological mechanisms underlying potential associations between COVID-19 vaccines and incident seizures remain unclear. Previous studies have suggested possible seizure risks associated with non-COVID-19 vaccines, including the measles, mumps, and rubella (MMR) vaccine, and the diphtheria and tetanus toxoids and whole-cell pertussis (DTP) vaccine [14,25]. These traditional vaccines utilize platforms such as live attenuated or inactivated vaccines, whereas the COVID-19 vaccines studied here employ newer platforms, including lipid nanoparticle-encapsulated nucleoside-modified RNA vaccines (BNT162b2, mRNA-1273) [26,27], viral vector vaccines (ChAdOx1 nCoV-19, Ad26.COV2.S) [28,29], or protein-based vaccines (NVX-CoV2373) [30]. Potential mechanisms hypothesized as seizure triggers—such as immune-mediated abnormal neuronal activity or sudden hyperthermia causing increased blood-brain barrier permeability during fever response [31,32] — are not inherently specific to any particular vaccine platform. Indeed, our study found a slightly increased NOS risk with mRNA-1273, which was not observed for the analogous nucleic acid-based vaccine, BNT162b2. Comparative vaccine efficacy studies have reported more frequent reactogenicity-related adverse events, such as fever—potential seizure triggers—with mRNA-1273 compared to BNT162b2 [33]. This difference could be attributed to significantly greater humoral immunogenicity (higher antibody titers) observed among mRNA-1273 recipients [34,35]. It is plausible that variations in immune responses that can trigger seizures might contribute to differences in observed seizure risks. However, given inconsistent findings from analyses stratified by mRNA-1273 doses, the possibility of a chance finding cannot be excluded, highlighting the need for further research.

In our study, individuals with a prior seizure diagnosis were excluded to accurately identify incident seizure cases. While this approach enhanced the precision of our NOS assessment, it concurrently limited our ability to assess the risk of recurrent seizures following vaccination among people with epilepsy (PWE). The clinical concern regarding potential exacerbation of seizure symptoms following COVID-19 vaccination in PWE warrants further investigation. Multiple studies to date have consistently indicated that COVID-19 vaccines are well-tolerated by individuals with pre-existing epilepsy [36-39]. The favorable safety profiles observed in these studies offer encouraging evidence supporting vaccine confidence among the PWE population. Additional studies involving large cohorts of individuals with epilepsy are necessary to provide robust safety data in this important clinical context.

Our study has several strengths. First, using a comprehensive nationwide database allowed us to derive precise risk estimates despite the rarity of the outcome. Over 100 million vaccine doses were administered during the study period, including booster doses and multiple vaccine types, enabling a thorough safety assessment complementary to previous studies. Second, the SCCS analysis facilitated vaccine safety evaluations within a population characterized by high COVID-19 vaccination rates, using within-individual comparisons that effectively controlled for time-invariant confounders. Third, subgroup analyses stratified by various underlying comorbidities further reinforced the safety of COVID-19 vaccines for the majority of the population.

However, several limitations should be acknowledged. First, the SCCS analysis did not control for time-varying confounders. Given the observational nature of our study, we cannot exclude the possibility of residual confounding and thus cannot infer causality from our results. Second, the use of claims data—relying on diagnosis codes and drug prescriptions to define NOS—did not allow differentiation of seizure types based on symptomatic etiology or epileptiform electroencephalography patterns (e.g., generalized seizures, tonic-clonic seizures, absence seizures, myoclonic seizures, or febrile seizures), each of which has distinct etiologies and risk factors [40]. Furthermore, defining NOS cases as seizure diagnoses accompanied by same-day ASM prescriptions could have underestimated true incident seizure occurrences. Lastly, our study only included Korean individuals, potentially limiting the generalizability of the findings to other ethnic groups.

In conclusion, this nationwide study found no evidence of an increased risk of NOS following COVID-19 vaccination. Although a marginally increased risk was observed with the mRNA-1273 vaccine, further research is necessary to investigate the observed differences in risk across vaccine types. Given the small absolute risk of NOS identified in this study, our findings provide valuable real-world evidence from a large population, contributing to enhanced public confidence in COVID-19 vaccination programs.

## Figures and Tables

**Figure f4-epih-47-e2025024:**
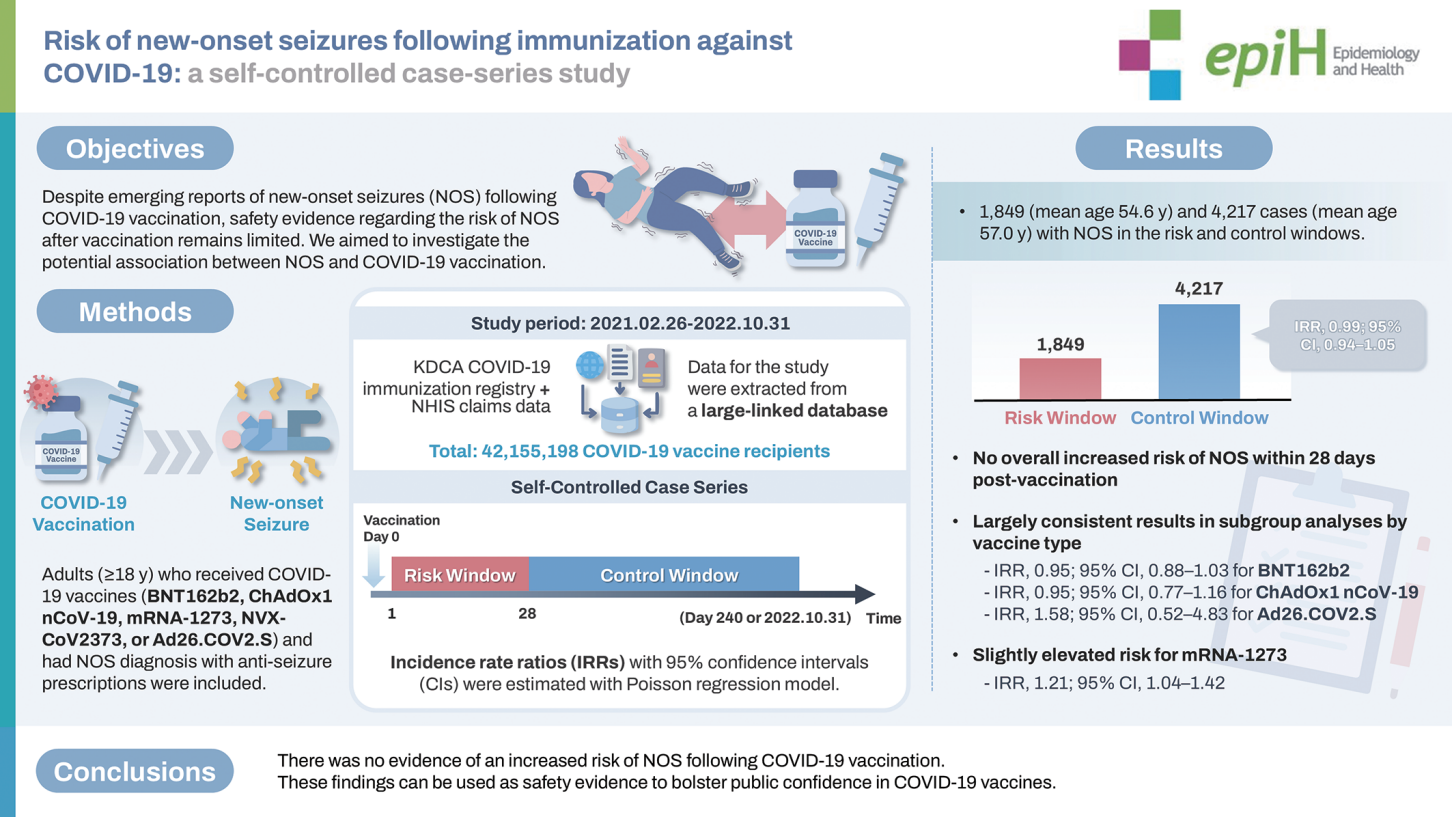


**Table 1.** Baseline characteristics of new-onset seizure cases following COVID-19 vaccination, stratified by exposure windows

Characteristics	Risk window (n=1,849)	Control window (n=4,217)	p-value
Age (yr)	54.6±19.7	57.0±19.9	<0.001
18-29	274 (14.8)	547 (13.0)	<0.001
30-39	203 (11.0)	381 (9.0)	
40-49	250 (13.5)	529 (12.5)	
50-59	295 (16.0)	712 (16.9)	
60-69	348 (18.8)	766 (18.2)	
70-79	273 (14.8)	617 (14.6)	
≥80	206 (11.1)	665 (15.8)	
Sex			0.405
Male	1,081 (58.5)	2,417 (57.3)	
Female	768 (41.5)	1,800 (42.7)	
Health insurance type			0.138
National Health Insurance	1,694 (91.6)	3,813 (90.4)	
Medical Aid	155 (8.4)	404 (9.6)	
Region of residence			0.409
Metropolitan	1,206 (65.2)	2,704 (64.1)	
Rural	643 (34.8)	1,513 (35.9)	
CCI	1.7±2.2	1.9±2.3	
<5	1,647 (89.1)	3,666 (86.9)	0.020
≥5	202 (10.9)	551 (13.1)	0.010
Comorbidities			
Myocardial infarction	21 (1.1)	54 (1.3)	0.638
Congestive heart failure	129 (7.0)	308 (7.3)	0.650
Peripheral vascular disease	256 (13.8)	609 (14.4)	0.541
Cerebrovascular disease	303 (16.4)	761 (18.0)	0.118
Dementia	255 (13.8)	715 (17.0)	0.002
Chronic pulmonary disease	284 (15.4)	739 (17.5)	0.038
Rheumatic disease	48 (2.6)	128 (3.0)	0.348
Peptic ulcer disease	289 (15.6)	738 (17.5)	0.074
Mild liver disease	394 (21.3)	934 (22.1)	0.467
Diabetes mellitus	384 (20.8)	951 (22.6)	0.123
Diabetic complications	146 (7.9)	326 (7.7)	0.825
Hemiplegia or paraplegia	56 (3.0)	160 (3.8)	0.139
Renal disease	76 (4.1)	178 (4.2)	0.843
Cancer	85 (4.6)	230 (5.5)	0.166
Serious liver disease	7 (0.4)	22 (0.5)	0.457
Solid/metastatic tumor	13 (0.7)	27 (0.6)	0.781
HIV infection	0 (0)	1 (0)	0.508

Values are presented mean±standard deviation or number (%). COVID-19, coronavirus disease 2019; CCI, Charlson comorbidity index; HIV, human immunodeficiency virus.

**Table 2.** Risk of new-onset seizures within 28 days following COVID-19 vaccination, stratified by age, sex, insurance type, region, and comorbidities

Variables	No. of event		Person-years		IR		IRR (95% CI)
	Risk	Control	Risk	Control	Risk	Control	
Age (yr)							
18-29	274	547	165.77	370.93	1.65	1.47	1.12 (0.97, 1.29)
30-39	203	381	113.67	278.76	1.79	1.37	1.31 (1.10, 1.55)
40-49	250	529	161.79	354.20	1.55	1.49	1.03 (0.89, 1.20)
50-59	295	712	213.46	457.74	1.38	1.56	0.89 (0.78, 1.02)
60-69	348	766	231.64	495.26	1.50	1.55	0.97 (0.86, 1.10)
70-79	273	617	175.58	400.41	1.55	1.54	1.01 (0.88, 1.16)
≥80	206	665	153.67	389.04	1.34	1.71	0.78 (0.67, 0.92)
Sex							
Male	1,081	2,417	706.52	1,586.99	1.53	1.52	1.00 (0.94, 1.08)
Female	768	1,800	509.06	1,159.35	1.51	1.55	0.97 (0.89, 1.06)
Insurance type							
National Health Insurance	1,694	3,813	1,105.49	2,496.18	1.53	1.53	1.00 (0.95, 1.06)
Medical Aid	155	404	110.09	250.16	1.41	1.61	0.87 (0.72, 1.05)
Region							
Metropolitan	1,206	2,704	784.34	1,766.73	1.54	1.53	1.00 (0.94, 1.07)
Rural	643	1,513	431.24	979.61	1.49	1.54	0.97 (0.88, 1.06)
CCI							
<5	1,647	3,666	1,071.25	2,410.55	1.54	1.52	1.01 (0.95, 1.07)
≥5	202	551	144.33	335.79	1.40	1.64	0.85 (0.73, 1.00)
History of myocardial infarction							
No	1,828	4,163	1,201.95	2,712.95	1.52	1.53	0.99 (0.94, 1.05)
Yes	21	54	13.63	33.39	1.54	1.62	0.95 (0.58, 1.57)
History of congestive heart failure							
No	1,720	3,909	1,132.71	2,552.71	1.52	1.53	0.99 (0.94, 1.05)
Yes	129	308	82.86	193.63	1.56	1.59	0.98 (0.80, 1.20)
History of peripheral vascular disease							
No	1,593	3,608	1,043.07	2,357.88	1.53	1.53	1.00 (0.94, 1.06)
Yes	256	609	172.51	388.46	1.48	1.57	0.95 (0.82, 1.09)
History of stroke							
No	1,546	3,456	1,012.51	2,271.09	1.53	1.52	1.00 (0.95, 1.07)
Yes	303	761	203.07	475.25	1.49	1.60	0.93 (0.82, 1.06)
History of dementia							
No	1,594	3,502	1,039.13	2,311.65	1.53	1.51	1.01 (0.95, 1.07)
Yes	255	715	176.45	434.69	1.45	1.64	0.88 (0.76, 1.01)
History of chronic pulmonary disease							
No	1,565	3,478	1,014.62	2,296.18	1.54	1.51	1.02 (0.96, 1.08)
Yes	284	739	200.96	450.17	1.41	1.64	0.86 (0.75, 0.99)
History of rheumatic disease							
No	1,801	4,089	1,179.00	2,665.23	1.53	1.53	1.00 (0.94, 1.05)
Yes	48	128	36.58	81.11	1.31	1.58	0.83 (0.60, 1.16)
History of peptic ulcer disease							
No	1,560	3,479	1,007.29	2,285.87	1.55	1.52	1.02 (0.96, 1.08)
Yes	289	738	208.29	460.47	1.39	1.60	0.87 (0.76, 0.99)
History of mild liver disease							
No	1,455	3,283	946.21	2,151.30	1.54	1.53	1.01 (0.95, 1.07)
Yes	394	934	269.37	595.04	1.46	1.57	0.93 (0.83, 1.05)
History of diabetes mellitus							
No	1,465	3,266	952.53	2,149.65	1.54	1.52	1.01 (0.95, 1.08)
Yes	384	951	263.05	596.69	1.46	1.59	0.92 (0.81, 1.03)
History of diabetic complications							
No	1,703	3,891	1,123.37	2,533.65	1.52	1.54	0.99 (0.93, 1.04)
Yes	146	326	92.21	212.69	1.58	1.53	1.03 (0.85, 1.25)
History of hemiplegia/paraplegia							
No	1,793	4,057	1,175.73	2,650.92	1.53	1.53	1.00 (0.94, 1.05)
Yes	56	160	39.85	95.42	1.41	1.68	0.84 (0.62, 1.14)
History of renal disease							
No	1,773	4,039	1,169.29	2,629.74	1.52	1.54	0.99 (0.93, 1.04)
Yes	76	178	46.29	116.61	1.64	1.53	1.08 (0.82, 1.41)
History of cancer							
No	1,764	3,987	1,150.92	2,606.63	1.53	1.53	1.00 (0.95, 1.06)
Yes	85	230	64.66	139.71	1.31	1.65	0.80 (0.62, 1.02)
History of serious liver disease							
No	1,842	4,195	1,209.39	2,732.67	1.52	1.54	0.99 (0.94, 1.05)
Yes	7	22	6.19	13.67	1.13	1.61	0.70 (0.30, 1.67)
History of solid/metastatic tumor							
No	1,836	4,190	1,208.19	2,731.41	1.52	1.53	0.99 (0.94, 1.05)
Yes	13	27	7.39	14.93	1.76	1.81	0.97 (0.52, 1.83)
History of HIV infection							
No	1,849	4,216	1,215.35	2,745.92	1.52	1.54	0.99 (0.94, 1.05)
Yes	0	1	0.23	0.42	N/A	2.37	N/A

COVID-19, coronavirus disease 2019; CCI, Charlson comorbidity index; CI, confidence interval; IR, incidence rate; IRR, incidence rate ratio; HIV, human immunodeficiency virus; N/A, not available.

## Supplementary materials

Supplementary Material 1. Illustration of self-controlled case series design assessing the risk of new-onset seizures following COVID-19 vaccinations.

Supplementary Material 2. Seizure case definition based on diagnostic and prescription codes

Supplementary Material 3. .STROBE Statement—checklist of items that should be included in reports of observational studies

Supplementary Material 4. Risk of seizure within 28 days of mRNA-1273, overall and stratified by dose, sex
