# Supplementary material for: Risk of new-onset seizures following immunization against COVID-19: a self-controlled case-series study
Source: Epidemiol Health. 2025 May 2;47:e2025024. doi: 10.4178/epih.e2025024 (PMC12425699; doi:10.4178/epih.e2025024)
Supplement: Supplementary Material 1. — Illustration of self-controlled case series design assessing the risk of new-onset seizures following COVID-19 vaccinations. [file epih-47-e2025024-Supplementary-1.docx]

**Supplementary Material 1.** Illustration of self-controlled case series design assessing the risk of new-onset seizures following COVID-19 vaccinations.

**
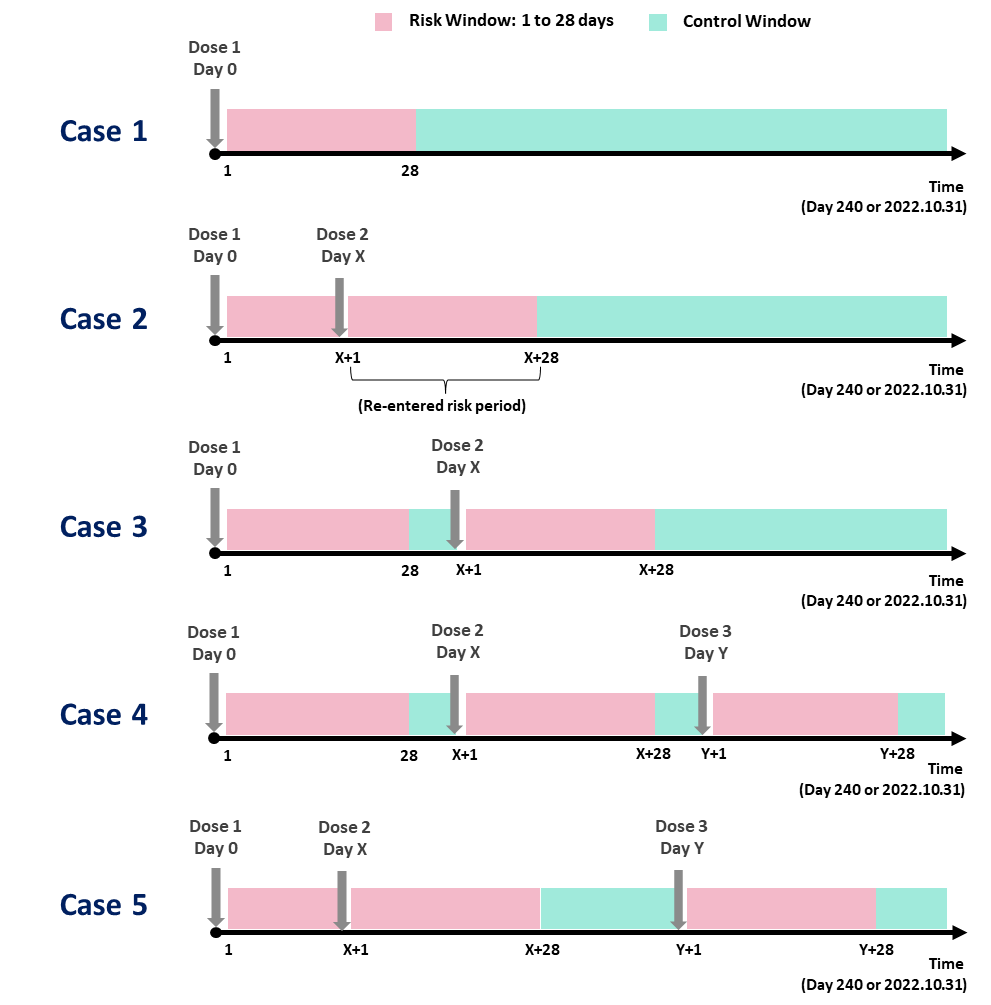
**
