# Supplementary material for: Risk of new-onset seizures following immunization against COVID-19: a self-controlled case-series study
Source: Epidemiol Health. 2025 May 2;47:e2025024. doi: 10.4178/epih.e2025024 (PMC12425699; doi:10.4178/epih.e2025024)
Supplement: Supplementary Material 2. — Seizure case definition based on diagnostic and prescription codes [file epih-47-e2025024-Supplementary-2.docx]

**Supplementary Material 2**. Seizure case definition based on diagnostic and prescription codes

| **Diagnosis** | **ICD-10 code** |
| --- | --- |
| Epilepsy | G40 |
| Status epilepticus | G41 |
| Convulsion, not elsewhere classifiable | R56 |
| Dissociative convulsions | F44.5 |
| **Anti-seizure medications** | **WHO-ATC code** |
| Phenobarbital, primidone | N03AA |
| Phenytoin, fosphenytoin | N03AB |
| Paramethadione, trimethadione, ethadione | N03AC |
| Ethosuximide | N03AD |
| Clonazepam | N03AE |
| Carbamazepine, oxcarbazepine, rufinamide, eslicarbazepine | N03AF |
| Valproic acid, vigabatrin | N03AG |
| Lamotrigine, topiramate, levetiracetam, zonisamide, stiripentol, lacosamide, perampanel, brivaracetam, cannabidiol | N03AX |

**Abbreviations**: ATC, anatomical therapeutic classification; ICD, international classification of diseases; WHO, world health organization
