# Supplementary material for: Risk of new-onset seizures following immunization against COVID-19: a self-controlled case-series study
Source: Epidemiol Health. 2025 May 2;47:e2025024. doi: 10.4178/epih.e2025024 (PMC12425699; doi:10.4178/epih.e2025024)
Supplement: Supplementary Material 3. — .STROBE Statement—checklist of items that should be included in reports of observational studies [file epih-47-e2025024-Supplementary-3.docx]

Supplementary Material 3.STROBE Statement—checklist of items that should be included in reports of observational studies

|  | | Item No | Recommendation |
| --- | --- | --- | --- |
| **Title and abstract** | | 1 | (*a*) Indicate the study’s design with a commonly used term in the title or the abstract  *“Title: Risk of New-Onset Seizures Following Immunization against COVID-19: A Self-Controlled Case-Series Study”* |
|  |  |  | (*b*) Provide in the abstract an informative and balanced summary of what was done and what was found  *“Balanced summary of methods and results with conclusions were presented in the structured Abstract.”* |
| Introduction | | | |
| Background/rationale | | 2 | Explain the scientific background and rationale for the investigation being reported  *“Background and rationale for the study were described in the Introduction.”* |
| Objectives | | 3 | State specific objectives, including any prespecified hypotheses  *“This study aimed to assess the association between the risk of new-onset seizures (NOS) and immunization against COVID-19 with self-controlled case series (SCCS) analysis.”* |
| Methods | | | |
| Study design | | 4 | Present key elements of study design early in the paper  *“Key elements of study design including data source, study population, study design, exposure, outcome was described throughout the Method.”* |
| Setting | | 5 | Describe the setting, locations, and relevant dates, including periods of recruitment, exposure, follow-up, and data collection  *“Setting, study periods, exposure, follow-up, data source were described throughout the Method.”* |
| Participants | | 6 | (*a*) *Cohort study*—Give the eligibility criteria, and the sources and methods of selection of participants. Describe methods of follow-up  *Case-control study*—Give the eligibility criteria, and the sources and methods of case ascertainment and control selection. Give the rationale for the choice of cases and controls  *“Eligibility criteria (inclusion/exclusion criteria for study population) for self-controlled case study analysis has been described throughout the Method.”*  *Cross-sectional study*—Give the eligibility criteria, and the sources and methods of selection of participants |
|  |  |  | (*b*) *Cohort study*—For matched studies, give matching criteria and number of exposed and unexposed  *Case-control study*—For matched studies, give matching criteria and the number of controls per case |
| Variables | | 7 | Clearly define all outcomes, exposures, predictors, potential confounders, and effect modifiers. Give diagnostic criteria, if applicable  *“All outcomes, exposures, and confounders have been defined in the Method.”* |
| Data sources/ measurement | | 8* | For each variable of interest, give sources of data and details of methods of assessment (measurement). Describe comparability of assessment methods if there is more than one group  *“Details of variable of interest and methods of assessment were described in the Method.”* |
| Bias | | 9 | Describe any efforts to address potential sources of bias  *“Efforts to address potential bias were described in the Method.”* |
| Study size | | 10 | Explain how the study size was arrived at  *“Not applicable”* |
| Quantitative variables | | 11 | Explain how quantitative variables were handled in the analyses. If applicable, describe which groupings were chosen and why  “Age groups were stratified by 10-year band.” |
| Statistical methods | | 12 | (*a*) Describe all statistical methods, including those used to control for confounding  *“Statistical methods were described in the Method.”* |
|  |  |  | (*b*) Describe any methods used to examine subgroups and interactions  *“Subgroup analyses were described in the Method.”* |
|  |  |  | (*c*) Explain how missing data were addressed  *“Missing or incomplete data were excluded from the study cohort.”* |
|  |  |  | (*d*) *Cohort study*—If applicable, explain how loss to follow-up was addressed  *Case-control study*—If applicable, explain how matching of cases and controls was addressed  *Cross-sectional study*—If applicable, describe analytical methods taking account of sampling strategy  *“Not applicable”* |
|  |  |  | (*e*) Describe any sensitivity analyses  *“Sensitivity analyses were described in the Method.”* |
| Results | | | |
| Participants | 13* | (a) Report numbers of individuals at each stage of study—eg numbers potentially eligible, examined for eligibility, confirmed eligible, included in the study, completing follow-up, and analysed  *“Reported in the Results with flowchart in eFigure 1.”* | |
|  |  | (b) Give reasons for non-participation at each stage  *“Exclusion criteria was described in the flowchart in eFigure 1.”* | |
|  |  | (c) Consider use of a flow diagram  *“Study population flowchart was presented in eFigure 1.”* | |
| Descriptive data | 14* | (a) Give characteristics of study participants (eg demographic, clinical, social) and information on exposures and potential confounders  *“Described in the first paragraph in the Results.”* | |
|  |  | (b) Indicate number of participants with missing data for each variable of interest  *“Missing or incomplete data were excluded from the study cohort.”* | |
|  |  | (c) *Cohort study*—Summarise follow-up time (eg, average and total amount) | |
| Outcome data | 15* | *Cohort study*—Report numbers of outcome events or summary measures over time | |
|  |  | *Case-control study—*Report numbers in each exposure category, or summary measures of exposure  *“Number of events in each exposure category were presented in Figure 2 and described in the Results.”* | |
|  |  | *Cross-sectional study—*Report numbers of outcome events or summary measures | |
| Main results | 16 | (*a*) Give unadjusted estimates and, if applicable, confounder-adjusted estimates and their precision (eg, 95% confidence interval). Make clear which confounders were adjusted for and why they were included  *“Point estimates of IRR and 95% confidence intervals for all outcomes were described in Figure 2 and the Results.”* | |
|  |  | (*b*) Report category boundaries when continuous variables were categorized  *“Described in the Results.”* | |
|  |  | (*c*) If relevant, consider translating estimates of relative risk into absolute risk for a meaningful time period  *“Absolute number of cases and incidence rates were described in the Results.”* | |
| Other analyses | 17 | Report other analyses done—eg analyses of subgroups and interactions, and sensitivity analyses  *“Results for the subgroup and sensitivity analyses were described in the Results.”* | |
| Discussion | | | |
| Key results | 18 | Summarise key results with reference to study objectives  *“Key results were summarized in the first paragraph of the Discussions.”* | |
| Limitations | 19 | Discuss limitations of the study, taking into account sources of potential bias or imprecision. Discuss both direction and magnitude of any potential bias  *“Limitations of the study were described in the 6th paragraph of the Discussions.”* | |
| Interpretation | 20 | Give a cautious overall interpretation of results considering objectives, limitations, multiplicity of analyses, results from similar studies, and other relevant evidence  *“Interpretation for the results were elaborated throughout the Discussions.”* | |
| Generalisability | 21 | Discuss the generalisability (external validity) of the study results  *“Generalizability of the study results were described in the Discussions.”* | |
| Other information | | | |
| Funding | 22 | Give the source of funding and the role of the funders for the present study and, if applicable, for the original study on which the present article is based  *“Disclosures including funding source were described.”* | |

*Give information separately for cases and controls in case-control studies and, if applicable, for exposed and unexposed groups in cohort and cross-sectional studies.

**Note:** An Explanation and Elaboration article discusses each checklist item and gives methodological background and published examples of transparent reporting. The STROBE checklist is best used in conjunction with this article (freely available on the Web sites of PLoS Medicine at http://www.plosmedicine.org/, Annals of Internal Medicine at http://www.annals.org/, and Epidemiology at http://www.epidem.com/). Information on the STROBE Initiative is available at www.strobe-statement.org.
