# Supplementary material for: Risk of new-onset seizures following immunization against COVID-19: a self-controlled case-series study
Source: Epidemiol Health. 2025 May 2;47:e2025024. doi: 10.4178/epih.e2025024 (PMC12425699; doi:10.4178/epih.e2025024)
Supplement: Supplementary Material 4. — Risk of seizure within 28 days of mRNA-1273, overall and stratified by dose, sex [file epih-47-e2025024-Supplementary-4.docx]

**Supplementary Material 4**. Risk of seizure within 28 days of mRNA-1273, overall and stratified by dose, sex

|  | **No. of event** | | **Person-years** | | **IR** | | **IRR  (95% CI)** |
| --- | --- | --- | --- | --- | --- | --- | --- |
|  | **Risk** | **Control** | **Risk** | **Control** | **Risk** | **Control** |  |
| **Overall** | 237 | 424 | 140.02 | 303.50 | 1.69 | 1.40 | 1.21 (1.04-1.42) |
| 1st dose | 78 | 424 | 55.22 | 303.50 | 1.41 | 1.40 | 1.01 (0.79-1.29) |
| 2nd dose | 105 | 398 | 54.20 | 276.38 | 1.94 | 1.44 | 1.35 (1.09-1.67) |
| 3rd dose | 54 | 94 | 30.60 | 68.62 | 1.76 | 1.37 | 1.29 (0.92-1.80) |
| 4th dose | 0 | 0 |  |  | N/A | N/A | N/A |
| 1st/2nd dose | 183 | 424 | 109.42 | 303.50 | 1.67 | 1.40 | 1.20 (1.01-1.42) |
| **Sex** |  |  |  |  |  |  |  |
| Male | 137 | 267 | 85.81 | 184.66 | 1.60 | 1.45 | 1.10 (0.90-1.35) |
| Female | 100 | 157 | 54.21 | 118.84 | 1.84 | 1.32 | 1.40 (1.09-1.79) |

**Abbreviations**: CI, Confidence Interval; IR, Incidence Rate; IRR, Incidence Rate Ratio
